# Pathological Changes and Expression of JAK-STAT Signaling Pathway Hallmark Proteins in Rat Retinas at Different Time Points After Retinal Ischemia Reperfusion Injury

**DOI:** 10.3389/pore.2022.1610385

**Published:** 2022-04-19

**Authors:** Shun Wang, Aihua Yu, Mengyao Han, Xiaomin Chen, Zhi Li, Min Ke, Xiaojun Cai, Ming Ai, Yiqiao Xing

**Affiliations:** ^1^ Eye Center, Renmin Hospital of Wuhan University, Wuhan, China; ^2^ Department of Ophthalmology, Zhongnan Hospital of Wuhan University, Wuhan, China; ^3^ Retinal and Vitreous Diseases Department, Wuhan Aier Eye Hospital of Wuhan University, Wuhan, China

**Keywords:** apoptosis, pathology, retina ischemia reperfusion injury, retinal ganglion cells, Janus kinase signal transducer and activator of transcription

## Abstract

Retinal ischemia reperfusion injury (RIRI) is a conventional pathological process in various retinal vascular diseases. Many studies select only one specific time point to apply drugs and then assess the therapeutic effect of drugs; however, the baselines are not the same at different time points, which may cause variation in the judgement. Therefore, further investigation is needed. Accordingly, this study aimed to investigate the pathological changes of retinal structure, expression of JAK-STAT signaling pathway hallmark proteins, and apoptosis at different time points after retinal ischemia reperfusion injury in rats. Sixty-six male SPF Sprague-Dawley rats were randomly divided into six groups: control group, RIRI 0, 6-, 24-, 72-, and 144-h groups. RIRI models were induced by perfusing equilibrium solution into the right eye anterior chamber to increase intraocular pressure to 110 mmHg for 60 min. Rats were sacrificed at different time points after reperfusion. Then hematoxylin-eosin staining, transmission electron microscope, immunohistochemistry, western blot, and TUNEL were used. Hematoxylin-eosin showed the pathological changes while transmission electron microscope revealed the ultra-structure changes of retina after RIRI. Immunohistochemistry showed that JAK2, STAT3, p-JAK2, p-STAT3, Bax, and Bcl-2 proteins mainly located in ganglion cell layer and inner nuclear layer, the relative expression of former five proteins had significant differences vs. control group (*p* < 0.05), while Bcl-2 had no significant difference. In western blot, the protein expressing of JAK2, STAT3, p-JAK2, p-STAT3, p-Akt, and Bax had significant differences vs. control group (*p* < 0.05), while Akt and Bcl-2 had no significant differences. TUNEL staining showed the number of apoptosis positive cells rose initially but declined later, with a peak value at RIRI 24 h group. The dynamic changes of hallmark proteins at different time points after RIRI indicate that JAK-STAT signaling pathway activates rapidly but weakens later and plays a vital role in RIRI, and apoptosis is involved in RIRI with a peak value at 24 h in the process, suggesting a potential therapeutic direction and time window for treating RIRI.

## Introduction

Retinal ischemia reperfusion injury (RIRI) is a conventional pathological process in various retinal vascular diseases, such as glaucoma, retinal vascular obstruction, diabetic retinopathy, and so on, which can cause serious visual dysfunction, and the mechanism of RIRI is sophisticated (1, 2). Janus kinase signaling transducer and activator of transcription (JAK-STAT) signaling pathway is a multifunctional signaling transcript channel that is involved in various biological processes, including cell proliferation, differentiation, apoptosis, immune regulation, and so on (3, 4). There is evidence that JAK-STAT signaling is a critical transducer in cardiomyocytes hypoxia injury (5), acute lung injury (6), cancer (7, 8, 9), rheumatoid arthritis (10), hematopoiesis and leukemia (11), obesity and diabetes (12, 13), and central nervous system (CNS) (14, 15) + disorders. In anatomy and tissue development, the retina is considered to be an extension of the CNS. It consists of retinal ganglion cells (RGC), whose axons form the optic nerve, and its fibers are actually axons of the CNS (16). However, the role of JAK-STAT signaling pathway in RIRI remains elusive and controversial. Most studies are mostly conducted through drug intervention, and many studies select only one specific time point, such as 0, 6, 24, 48, 72, or 144 h after RIRI to apply drugs and then assess the therapeutic effect of drugs. But in fact, the baselines are not the same at different time points (17), which may cause variation in the judgement. Therefore, further investigation is needed to thoroughly elucidate the continuous changes of JAK-STAT signaling pathway at different time points after RIRI in rat model. Accordingly, we researched the pathological structure, protein expression changes and effect of JAK-STAT signaling pathway at different time points of RIRI in this study to provide an intact baseline.

## Materials and Methods

### Animals

All animal experiments were carried out according to the National Institutes of Health guide for the care and use of Laboratory animals (NIH Publications No. 8023, revised 1978), conformed to the provisions of the Declaration of Helsinki (as revised in Edinburgh 2000). All animal handling protocols are in accordance with ARVO Statement for the Use of Animals in Ophthalmic and Vision Research. This study was approved by the Experimental Animal Welfare and Ethics, Zhongnan Hospital of Wuhan University (Aproval NO. ZN2021045).

Sprague-Dawley (SD) rats were the classical choice for RIRI model because they had good resistance to disease. Because rats of different sex had different sensitivity and stress response to RIRI, we choose all male rats to minimize the difference. Sixty-six healthy male specific pathogen-free (SPF) SD rats (250–300 g) were maintained in temperature-controlled rooms with a 12 h light/dark cycle with free access to food and water. We cleaned the cage every other day. After an adaptation of 1-week, the rats were divided into six groups randomly according to random number table: control group (CG), RIRI 0, 6-, 24-, 72-, and 144-h groups. In all six groups, there were three rats prepared for retinal sections of hematoxylin-eosin, immunohistochemistry, and terminal-deoxynucleoitidyl transferase mediated nick end labeling (TUNEL), and six rats for Western blot because the retinas were very little and consumed quickly when loading samples. There were an additional three rats for transmission electron microscopy in RIRI 72 h group, which was the most typical to reflect RIRI pathological and ultra-structure changes. The rats with normal ophthalmology and general condition before and after experiments were adopted in the study. And there were nine rats excluded because of unsuccessful RIRI, vitreous bleeding, anterior chamber piercing, endophthalmitis, or death from anaesthesia.

### RIRI Models

RIRI models were induced in the right eyes of the rats following previous report (18). The rats were anesthetized intraperitoneally with 40 mg/kg 1% pentobarbital sodium. The right eyes were subjected to topical anesthesia with 0.5% Proparacaine Hydrochloride (ALCON-COUVREUR n.v., Puurs) and tropicamide-phenylephrine eyedrops (Santen Pharmaceutical Cor., Ltd. Japan) to achieve mydriasis. Then a sterilized 30-gauge needle connected to equilibrium solution bottle was punctured into the right eye anterior chamber through cornea limbal, and the infusion bottle was raised to 150 cm height, which increased the intraocular pressure (IOP) to 110 mmHg (1 mmHg = 0.133 kPa). The conjunctiva and iris vessels turned white rapidly, the cornea experienced edema, and retinal pallor was observed by direct ophthalmoscope. During the infusion, levofloxacin eye gel was given to moisten the eye surface and prevent infection. After continuous high IOP for 60 min, the infusion needle was pulled out, the cornea, iris, and bulbar conjunctiva quickly recovered to normal, and the retina returned to orange-red color. In the control group, the same sterilized 30-gauge needle was penetrated into the anterior chamber, but removed immediately without perfusing solution. Rats in the same group were treated continuously to minimize potential confounders.

The RIRI rats were sacrificed after complete anesthesia at 0, 6, 24, 72, or 144 h after reperfusion, and the eyeballs were enucleated. The changes of retina pathological structure and ultrastructure were observed by hematoxylin-eosin staining and transmission electron microscopy. The protein distribution location and relative expression intensity of JAK2, phosphorylated JAK2 (p-JAK2), STAT3, phosphorylated STAT3 (p-STAT3), protein kinase B (PKB/Akt), phosphorylated Akt (p-Akt), Bcl-2, and Bax were tested by immunohistochemistry and Western Blot. The apoptosis of retina cells was assessed by TUNEL. The section reader was blind to the groups to avoid biases when collecting data.

### Hematoxylin-Eosin Staining

The retinal sections were stained with hematoxylin and eosin according to protocol (19). The slices through optic nerve were observed and photographed under an optical microscope (OLYMPUS, CX-21, Japan). The RGCs numbers were counted in a retinal length of 200 µm. The thickness of inner nuclear layer (INL) and retinal neuroepithelial layer (from the inner limiting membrane to photoreceptor layer) were measured in five different visual fields. The section reader was blind to the groups to avoid biases.

### Transmission Electron Microscope

The samples were prepared according to the improved method (20). Precooled 4°C physiological saline was rapidly perfused into the left ventricle, and then 200 ml fixation fluid of 2.5% glutaraldehyde and 4% paraformaldehyde was immediately injected. The eyeballs were enucleated, the retinas were peeled off and sheared into small blocks of 1 mm*1 mm*1 mm, fixed, soaked and embedded in epoxy resin Epon 812, cut into ultrathin section, stained by uranium acetate and leadcitrate, and observed by TEM (FEI Tecnai G2 F20, Netherlands).

### Immunohistochemistry

The paraffin retinal sections were processed according to IHC protocol and previous study (17). Photos were taken under an optical microscope (OLYMPUS, CX-21, Japan). Primary antibodies were as follows: JAK2 (bs-23003r, Bioss), STAT3 (60199-1-lg, Proteintech), p-JAK2 (ab32101, Abcam), p-STAT3 (sc-8059, Santa), Bcl-2 (ab194583, Abcam), and Bax (A19684, ABclonal). HRP-conjugated secondary antibodies were AS-1107 and AS-11069 (Aspen).

### Western Blot

The fresh retina was peeled meticulously on ice. Protein samples were added in SDS-PAGE gel to process electrophoresis following previous study (17). The blots were measured by AlphaEase FC software (Alpha Innotech, United States). Primary antibodies were as follows: JAK2 (#3230, CST), p-JAK2 (#3776, CST), STAT3 (ab68153, Abcam), p-STAT3 (ab76315, Abcam), Akt (#9272, CST), p-Akt (#4060, CST), Bcl-2 (ab196495, Abcam), Bax (#2772, CST), and β-Actin (TDY051, TDY). Secondary antibody was HRP-Goat anti Rabbit AS1107 (ASPEN).

### Terminal-Deoxynucleoitidyl Transferase Mediated Nick End Labeling Staining

TUNEL staining was performed on retinal sections with a detection kit (11684817910, Roche, Shanghai, China) according to the manufacturer’s instructions. DAPI was applied to mark the cell nucleus. The sections were photographed by a confocal laser scanning microscope (FV1200, Olympus, Tokyo, Japan). TUNEL-positive cells were counted by ImageJ software (National Institutes of Health, United States).

### Statistical Analysis

Statistical analyses were proceeded with SPSS 18.0 (SPSS, IL, United States). The results were shown as mean ± standard error of mean (S.E.M). The student’s t-test was used for normally distributed data of comparison between two groups, Mann-Whitney U test was adopted in skewed distribution. Ordinary one-way ANOVA was used for comparison between groups. The confidence interval was set at 95%, *p* < 0.05 was considered statistically significant. The first authors were aware of the group during the allocation and conduction of the experiment, but blind during the outcome assessment.

## Results

### HE Staining Results of Retinal Pathological Structure Changes After RIRI

**FIGURE 1:**
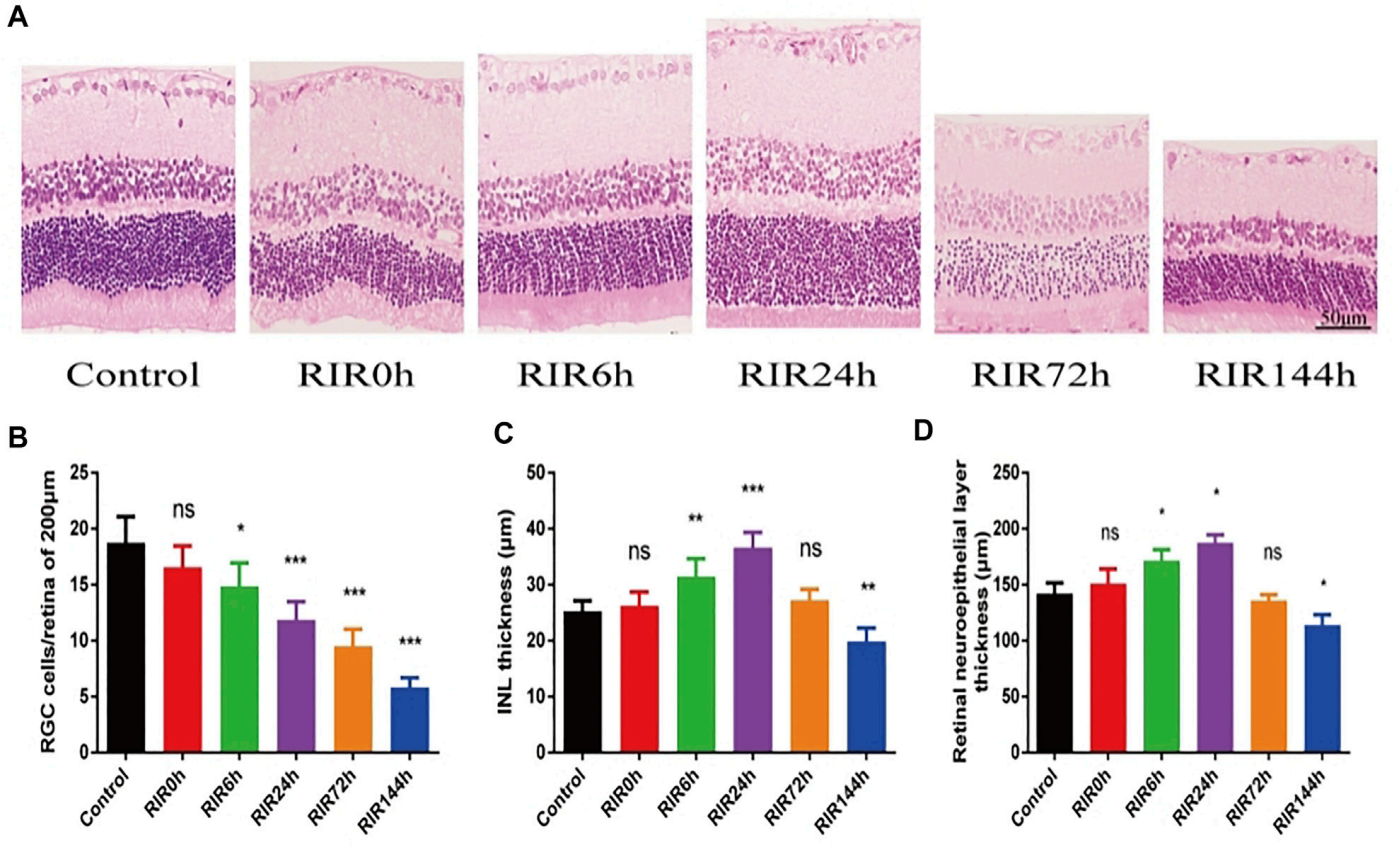
Pathological structure changes of retina in control, RIRI 0, 6, 24, 72, and 144 h groups by HE. **(A)** HE staining sections of retina (400 ×, scale bar = 50 μm). **(B)** RGC numbers of 200 μm length retina. **(C)** Thickness of INL. **(D)** Thickness of retinal neuroepithelial layer. N = 5 sections for measuring per group. Compared with control group; ns, no significance, *p < 0.05, **p < 0.01, ***p < 0.001.

We investigated the pathological structure changes of retina at different time points after RIRI through HE staining (Figure 1A, 400×). In the control group, the retinal surface was smooth, the structure in every layer was complete and clear, and the cells were arranged neatly. In RIRI groups, retinal edema was seen in the early stage, with a peak value in RIRI 24 h group, then the retina atrophied gradually in RIRI 72 and 144 h groups. The RGCs/200 µm retina were counted in six groups (Figure 1B). Compared with the control group, there was no significance in RIRI 0 h group, but significance in all other groups. The thickness of INL (Figure 1C) and retinal neuroepithelial layer (Figure 1D) had similar trends: compared with the control group, there were no statistical significance in RIRI 0 and 72 h groups or significant changes in RIRI 6, 24, and 144 h groups.

### TEM Results of Retinal Ultra-Structural Changes After RIRI

**FIGURE 2:**
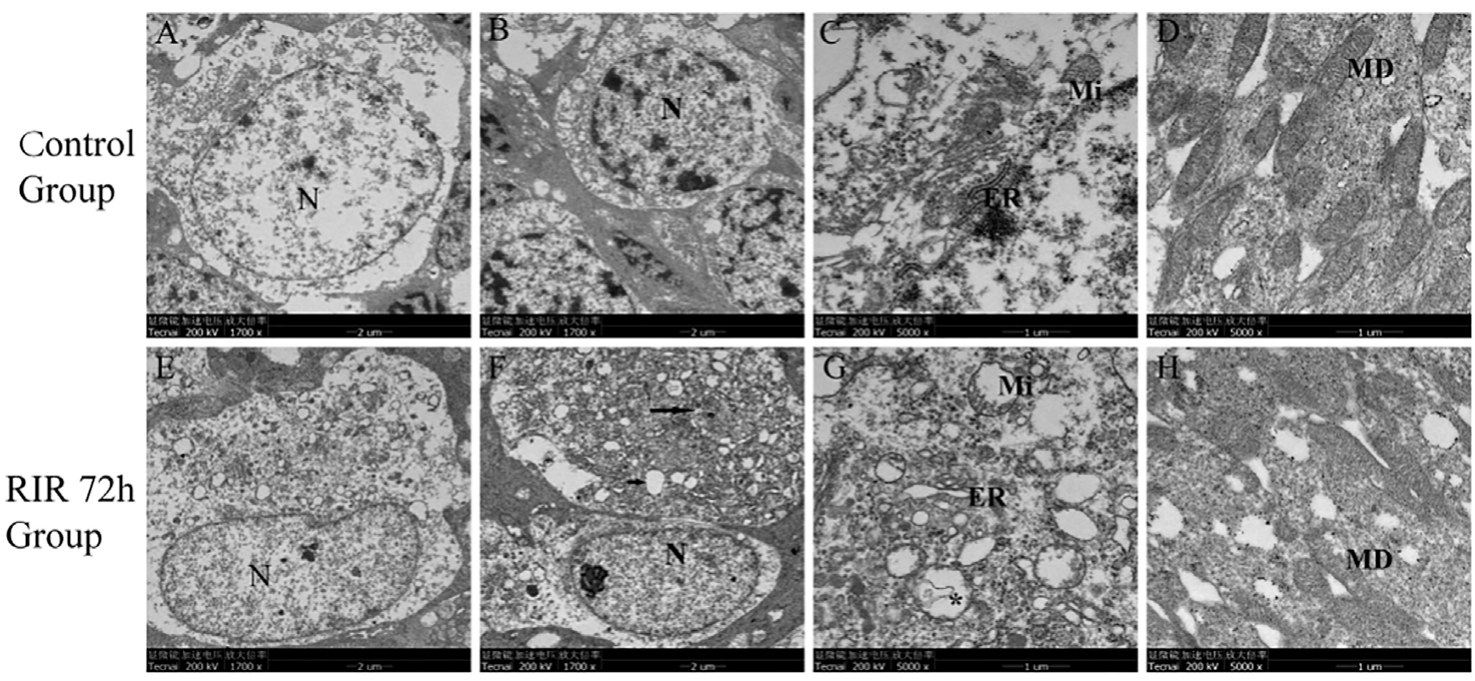
Ultrastructural changes of retina in control and RIRI 72 h groups by TEM. **(A–D)** Control group. **(E–H)** RIRI 72 h group. N, nucleus; ER, endoplasmic reticulum; Mi, mitochondrion; MD, membranous disc; long black arrow, apoptosis bodies; short black arrow, vacuoles; *, autolysosomes. Scale bars = 2 μm in Figure **(A,B,E,F)**, scale bars = 1 μm in Figure **(C,D,G,H)**.

Next, we looked into the ultra-structural changes of retina by TEM. In control group, the cell membrane and nuclear membrane were intact, the nucleus (N) was large and round (Figure 2A), the chromatin was evenly distributed in nucleus (Figure 2B), the mitochondrion (Mi) and endoplasmic reticulum (ER) were observed without swelling (Figure 2C), few cytoplasmic vacuoles and apoptotic bodies were observed in cytoplasm, and the specific membranous disc (MD) was complete and orderly arranged in photoreceptor layer (Figure 2D). In RIRI 72 h group, chromatin condensation and darkening (Figures 2E,F) were seen in the nucleus (N) and cytoplasmic vacuolation was seen in cytoplasm (Figures 2E–G). In the upper cell (Figure 2F), the nuclear membrane was dissolved, the cell organelle dispersed in cytoplasm, some apoptosis bodies (long black arrow) and plenty of vacuoles (short black arrow) were observed; in the inferior cell (Figure 2F), the cell membrane was not intact, with part of the cytoplasm overflowed. Mitochondrion (Mi) swelling, endoplasmic reticulum (ER) dilatation, and autolysosomes (*) formation were observed in cytoplasm (Figure 2G). Membranous disc (MD) degeneration in photoreceptor layer was observed (Figure 2H).

### Immunohistochemistry Staining Showing Protein Expression Localization and Relative Intensity of JAK2, STAT3, p-JAK2, p-STAT3, Bcl-2, and Bax in Retina After RIRI

**FIGURE 3:**
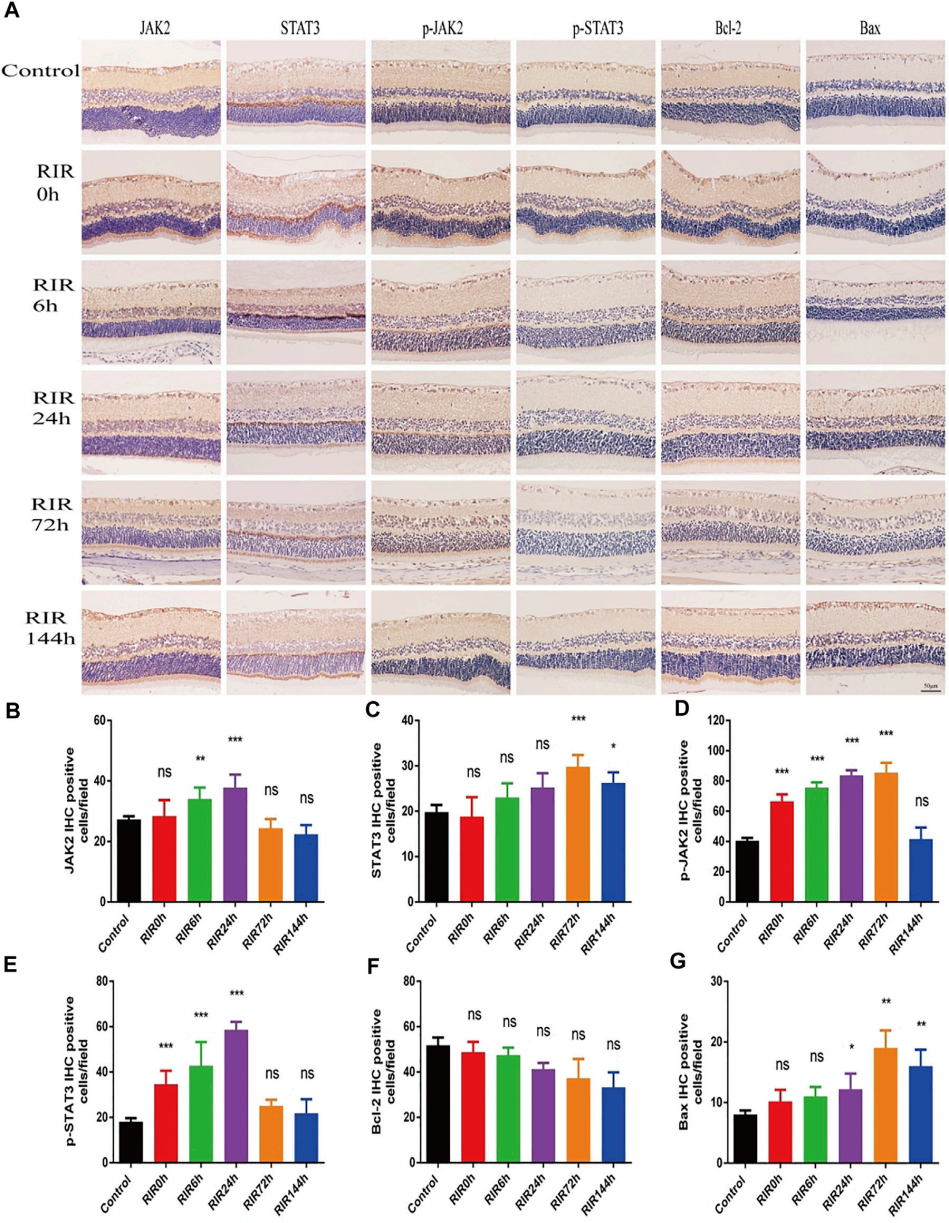
Immunohistochemistry staining showing protein expression and localization of JAK2, STAT3, p-JAK2, p-STAT3, Bcl-2, and Bax in control, RIRI 0, 6, 24, 72, and 144 h groups. **(A)** Protein expression of JAK2, STAT3, p-JAK2, p-STAT3, Bcl-2, and Bax in six groups. Scale bar = 50 μm. **(B–G)** Analysis of JAK2 **(B)**, STAT3 **(C)**, p-JAK2 **(D)**, p-STAT3 **(E)**, Bcl-2 **(F)**, and Bax **(G)** in six groups. N = 5 sections for measuring per group. Compared with control group; ns, no significance, *p < 0.05, **p < 0.01, ***p < 0.001.

IHC photos showed the protein expression location and relative intensity of JAK2, STAT3, p-JAK2, p-STAT3, Bcl-2, and Bax in control, RIRI 0, 6, 24, 72, and 144 h groups (Figure 3A). The positive proteins were dark yellow or brown blot stains in cells which were mainly located in retinal ganglion cell layer (GCL) and INL. As for the relative intensity, the numbers of IHC positive cells of JAK2, p-JAK2, STAT3, and p-STAT3 had similar trends: increased first and decreased later, with different peak values of significance compared with the control group (Figures 3B–E). There were no significant changes in the number of Bcl-2 positive cells in all RIRI groups compared with the control group (Figure 3F). However, the expression intensity of Bax had a significant rise in RIRI 24, 72, and 144 h groups compared with the control group (Figure 3G), indicating the enhancement of apoptosis at RIRI 24, 72, and 144 h time points.

### WB Protein Expression Changes of JAK-STAT Signaling Pathway After RIRI

**FIGURE 4:**
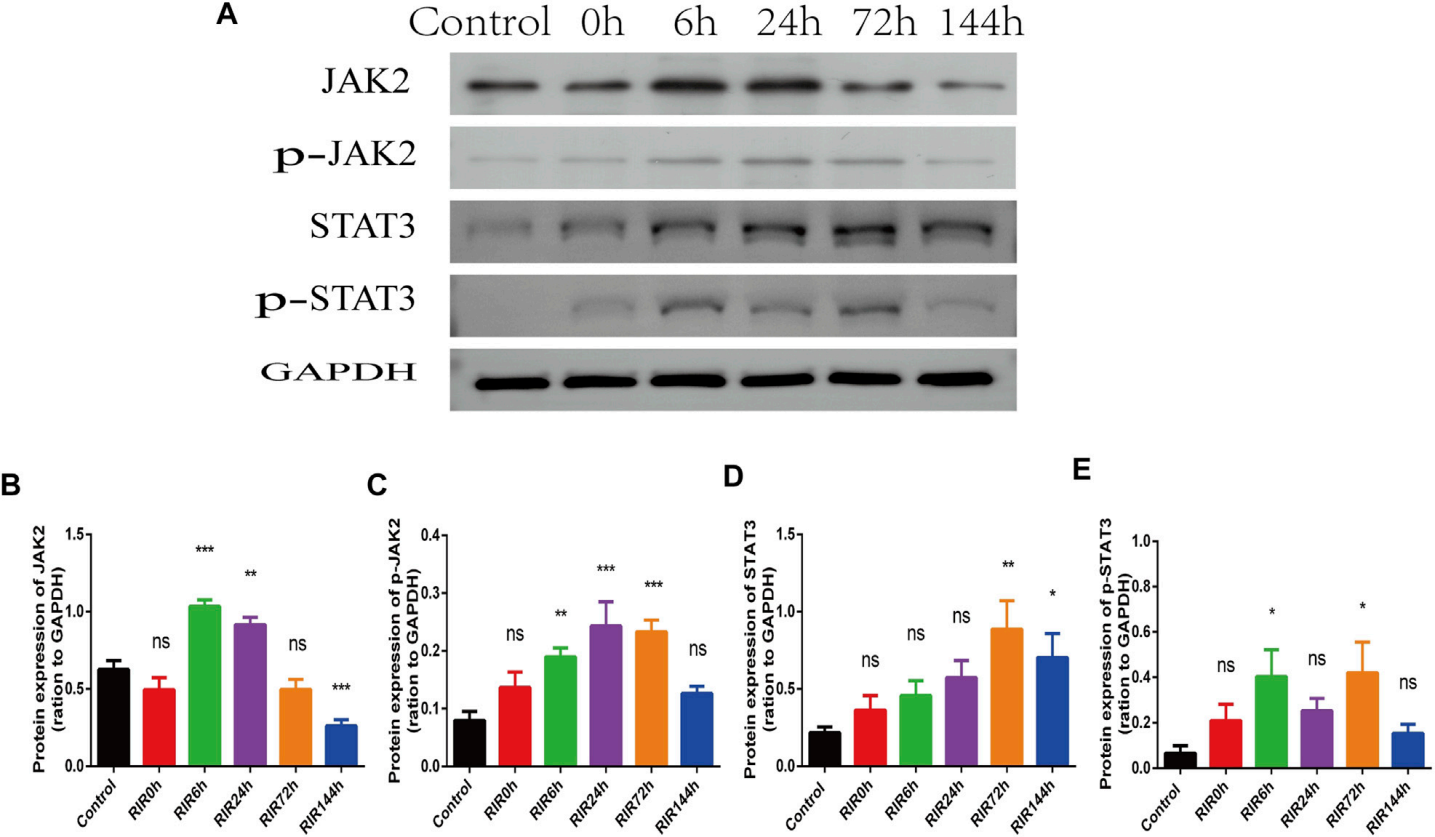
The hallmarker protein expression of JAK-STAT signaling pathway in control, RIRI 0, 6, 24, 72, and 144 h groups. **(A)** Protein expression of JAK2, p-JAK2, STAT3, and p-STAT3 in six time points groups. **(B–E)** Analysis of JAK2 **(B)**, p-JAK2 **(C)**, STAT3 **(D)**, and p-STAT3 **(E)** in six groups. N = 3–6 repeats per group. Compared with control group, ns, no significance, *p < 0.05, **p < 0.01, ***p < 0.001.

In WB results, the protein expression intensity of JAK2 and STAT3 (Figures 4A,B,D) had similar trends (increased first and decreased later). The activated protein p-JAK2 and p-STAT3 (Figures 4A,C,E) had weak expression in control groups, but increased immediately from RIRI 0 h, had significant differences between RIRI 6 h and 72 h, and then decreased gradually to have no significant difference in RIRI 144 h groups.

### Apoptosis in Retinal Cells After RIRI

**FIGURE 5:**
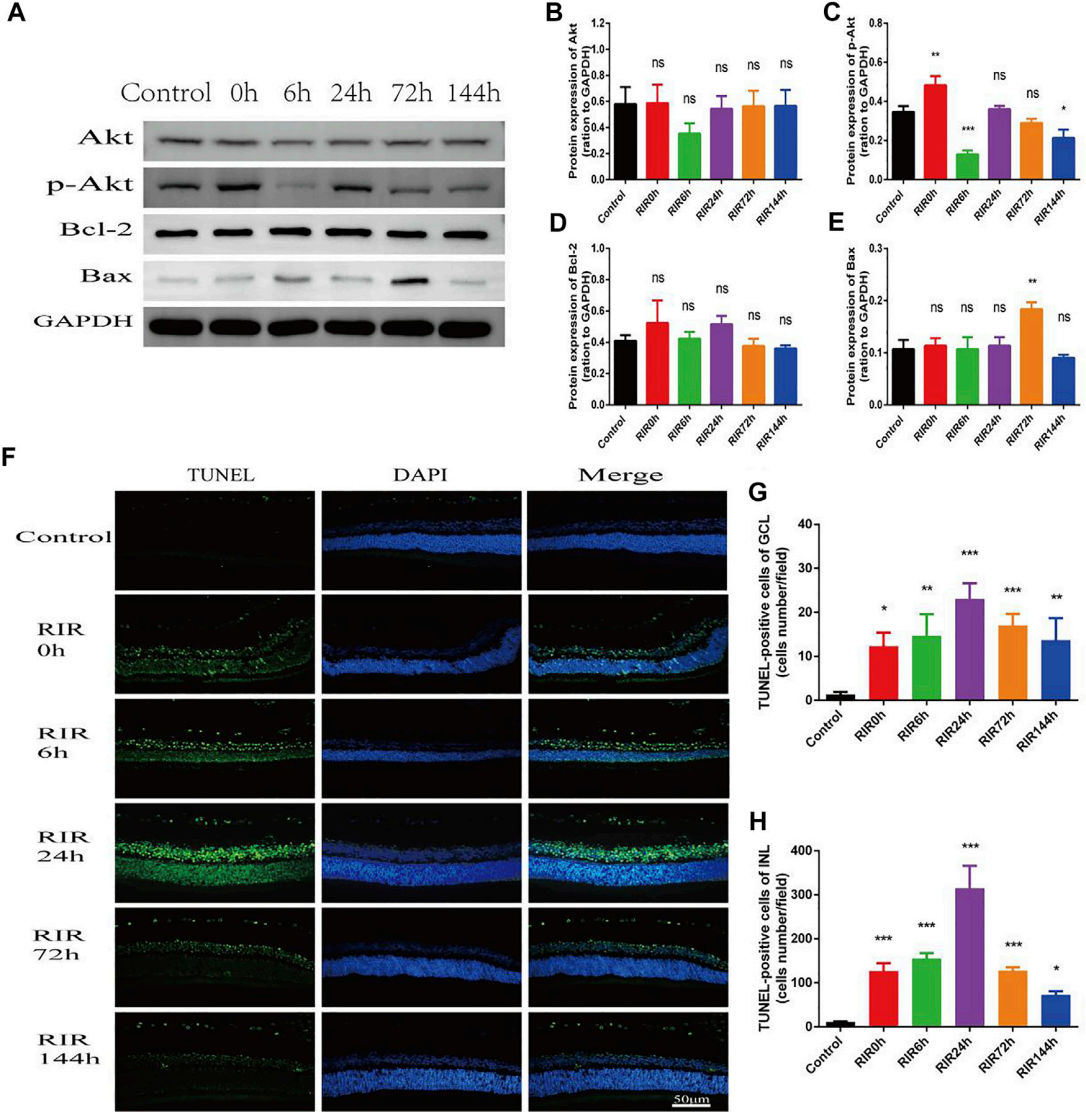
Apoptosis in control, RIRI 0, 6, 24, 72, and 144 h groups after RIRI. **(A)** Western blots of Akt, p-Akt, Bcl-2, and Bax. **(B–E)** Data analysis of Akt, p-Akt, Bcl-2, and Bax. **(F)** Retina sections were labeled with TUNEL (green) and DAPI (blue). Scale bar = 50 µm. **(G)** TUNEL-positive cells of GCL/field. **(H)** TUNEL-positive cells of INL/field. N = 3–6 repeats per group. Compared with control group, *p < 0.05, **p < 0.01, ***p < 0.001. GCL, ganglion cell layer; INL, inner nuclear layer.

Apoptosis was assessed by WB of apoptosis relative proteins and TUNEL staining in control, RIRI 0, 6, 24, 72, and 144 h groups (Figure 5). WB of proteins Akt, p-Akt, Bcl-2, and Bax were performed (Figure 5A). In total Akt (Figure 5B), there were no significant changes in all RIRI groups compared with control group; however, in p-Akt (Figure 5C), there was a significant increase in RIRI 0 h group (*p* < 0.05) and significant decrease in RIRI 6 and 144 h group (*p* < 0.05). The expression of Bcl-2 protein did not change dramatically in all RIRI groups (Figure 5D), while the expression of Bax increased significantly at RIRI 72 h group (Figure 5E), which confirmed that the apoptosis regulation system developed to promote apoptosis in RIRI.

In TUNEL staining, retina sections were labeled with TUNEL (green) and DAPI (blue). TUNEL positive cells were mainly located in GCL and INL (Figure 5F), and the numbers rose at first but declined later, with a peak value at RIRI 24 h group (Figures 5G,H). Compared with the control group, the TUNEL positive cells in both GCL and INL had remarkable statistic meaning in all RIRI groups (*p* < 0.05).

## Discussion

In clinical practice, RIRI exists in various retinal diseases, and it is a major cause leading to irreversible blindness (21). Rat transit high intraocular pressure model is a classical way to research RIRI (18, 22). We built the RIRI rat model successfully and explored the expression and effect of JAK-STAT signaling pathway proteins at different time points in RIRI subsequently.

First, we observed the retinal pathological structure and ultra-structure changes after RIRI through HE staining and TEM. The RGCs are the neurons in the retina that pass visual signaling to the visual cortex of brain (16). In HE staining, we found that the RGCs number decreased sharply after RIRI. The thickness of INL and retina neuroepithelial layer changed simultaneously, which indicated that the inner retina was sensitive to RIRI. Furthermore, we observed the delicate ultra-structure changes by TEM, and found that the micro-morphological structure changed obviously in RIRI, which was the basis of visual function loss.

Second, we evaluated the expression of JAK-STAT signaling pathway at different time points through the hallmark proteins JAK2, STAT3, p-JAK2, and p-STAT3 by WB and IHC. In WB results, JAK2, STAT3, p-JAK2, and p-STAT3 had similar trends: increased first and declined later, which recorded the start-up, up-regulation, and down-regulation of JAK-STAT signaling pathway. Furthermore, we observed the distribution location of these proteins and the relative intensity through IHC. However, the impact of JAK-STAT pathway is double-sided (23), and the results are not all the same in different studies and even in different assays. It may be because that JAK-STAT signaling pathway has complex crosstalk with quite a lot of other signaling pathways, such as mitogen-activated protein kinase (MAPK) pathway and phosphatidylinositol 3-kinase/protein kinase B (PI3K/Akt) pathway, which play similar or opposite roles in RIRI. Furthermore, stress response of animals or protein degradation of long-term storage may affect the expression. The dynamic changes of hallmark proteins at different time points indicate that JAK-STAT signaling pathway activates rapidly at an early stage and plays a vital role in RIRI.

Third, we assessed the apoptosis in RIRI by TEM, WB, and TUNEL staining. In TEM photos, plenty of vacuoles and some apoptosis bodies were observed in RIRI groups in contrast with the control group, which was direct evidence of apoptosis in RIRI (24). We performed WB of apoptosis proteins Akt, p-AKT, Bcl-2, and Bax. Akt is considered an important protein in apoptosis, and p-Akt is the activated form of Akt, which promotes the phosphorylation of downstream proteins (25). Bcl-2 and Bax are key target hallmarks in the apoptosis family (26–27). Bcl-2 is recognized as an anti-apoptotic protein, while Bax is a pro-apoptotic protein. In our WB results, the protein expression indicated the participation of apoptosis in RIRI. TUNEL staining provided further evidence of apoptosis, represented by protein location and relative quantitative changes. There were some differences between WB and TUNEL tests at the same time points, partly because WB results only showed the cross-sectional expression of limited apoptosis proteins at the present time point in RIRI, whereas TUNEL staining showed the accumulative apoptosis effect of different pathways and multiple proteins in the former RIRI duration. What’s more, we could speculate that there were many more pathways and proteins that participated in RIRI process than we previously thought, and even some converse effects happened and battled at the same time point to form a conclusive expression in the continuous duration of RIRI. The JAK-STAT signaling pathway is involved in apoptosis by regulating Bcl-2 and Bax, and partly by crosstalk with PI3K/AKT pathway. All this evidence confirms completely from various aspects that apoptosis is an important mechanism in RIRI.

In this study, we investigated the pathological changes of retinal structure, expression of JAK-STAT signaling pathway hallmark proteins, and apoptosis at different time points after retinal ischemia reperfusion injury in rats. Our study is limited by the rat model and small sample size. There are many drugs under research attempting to protect retina from RIRI (28–29), and JAK-STAT signaling pathway is the target for several diseases (30, 31). According to our study, JAK-STAT signaling pathway hallmark proteins are probably effective targets for the therapy of RIRI relative diseases. Specific inhibitors targeting these proteins may provide potential therapeutic strategies for RIRI and could provide further insights to other neurodegenerative diseases.

## Conclusion

JAK-STAT signaling pathway activates rapidly at an early stage but weakens later and plays a vital role in RIRI, and apoptosis is involved in RIRI with a peak value at 24 h in the process, suggesting a potential therapeutic direction and time window for treating RIRI.

## Data Availability

The original contributions presented in the study are included in the article/Supplementary Material, further inquiries can be directed to the corresponding authors.
